# Editorial: Editor's challenge: Dr. Qingxin Mu - how can nanomedicine approaches advance multi-targeting strategy in combination cancer therapy?

**DOI:** 10.3389/fonc.2024.1437497

**Published:** 2024-06-11

**Authors:** Marina Pinheiro, Qingxin Mu

**Affiliations:** ^1^ Department of Chemistry, Faculdade de Farmácia, Universidade do Porto, Porto, Portugal; ^2^ Public Health Unit, Local Health Unit Barcelos/Esposende, Barcelos, Portugal; ^3^ Department of Pharmaceutics, University of Washington, Seattle, WA, United States

**Keywords:** nanomedicine, nanoparticles, drug delivery, combination therapy, multi-targeted

Cancer remains a prominent global cause of death despite numerous advancements in prevention, diagnosis, and treatment approaches (1, 2). Combination therapy, which address multiple therapeutic targets, can exert pharmacological synergy and reduce dose-limiting toxicity of single agents, and have shown enhanced treatment outcomes in patients (3). However, owing to several challenges such as asynchronous circulation of drugs and lack of cancer cell specificity, the therapeutic synergy and overall outcomes of combination regimens may be suboptimal (4). Nanomedicine, as an innovative strategy in oncology research, has progressively matured over the past few decades. Currently, various nanomedicine approaches have been successfully utilized in both clinical and preclinical applications, ranging from imaging to drug delivery including combination therapeutic delivery, and have improved health outcomes (5–7). For example, Vyxeos® is a US FDA-approved nano-liposomal co-formulation of daunorubicin and cytarabine for combination treatment of secondary acute myeloid leukemia (8). Nonetheless, broad applications of nanomedicine approaches for improved combination therapy are still lacking. In this Research Topic entitled “Editor’s Challenge: Dr. Qingxin Mu – How can Nanomedicine Approaches Advance Multi-Targeting Strategy in Combination Cancer Therapy?”, we have gathered a few outstanding contributions highlighting nanomedicine as a new and promising strategy to improve combination therapy of cancer. The Research Topic contains 5 articles, namely 3 research articles, 1 review and 1 mini-review. The complete description of each study and the main results are presented in the full manuscripts and readers are pleasantly invited to explore the contents in full detail.

In the manuscript written by Donovan et al., the authors provided scientific evidence and demonstrated that when combining specific FOXM1 inhibitor RCM1 loaded in poly-beta-amino esters and folic acid-containing nanoparticle delivery with low doses of vincristine for treatment, increased rhabdomyosarcoma (RMS) cell apoptosis and decreased proliferation were observed *in vitro* compared to single drugs alone. The combination therapy was safe as demonstrated by liver metabolic panels using peripheral blood serum. The authors also used RNA-seq of dissected RMS tumors and identified Chac1 as a uniquely downregulated gene after the combination treatment. Lastly, through an RNA depletion assay, the authors verified that the knockdown of Chac1 in RMS cells *in vitro* recapitulated the effects of the combination therapy.

Salman-Javan et al. authored a manuscript showing that the use of PEGylated niosomal nanoparticles synthesized via the thin-film hydration method and co-loaded with metformin and silibinin improved the therapeutic efficiency in lung cancer cells through synergistic effects compared to the individual therapies. Mechanistically, with qRT-PCR assay, they found that the metformin-silibinin combination notably decreased in the expression of hTERT and BCL-2 genes, accompanied by an increase in BAX expression in the treated A549 lung cancer cells. More importantly, the suppression of hTERT and BCL-2 expression and the elevation of BAX gene expression were significantly heightened when A549 cells were treated with the drug combination encapsulated in PEGylated niosomal nanoparticles.

In the manuscript written by Kauser et al., the authors developed an *Artemisia absinthium* extract-loaded nanoformulations using N-isopropyl acrylamide, N-vinyl pyrrolidone, and acrylic acid-based polymeric nanoparticles. The authors employed nano LC-MS/MS to identify differences in secretory protein expression associated with the treatment of breast cancer cell lines (MCF-7 and MDA-MB-231) and performed several bioinformatics analyses. Their results demonstrated that the formulations were able to invade the breast cancer tumor microenvironment, transform the communication network between the cancer cells, affect potential drivers of microtubular integrity, nucleosome assembly, and cell cycle; and eventually cause cell death.

The manuscript of Liao et al. highlighted the autophagy-mediated nanomaterials’ therapeutic efficacy and potential applications. The autophagy signaling pathway for tumor therapy was reviewed, including oxidative stress, mammalian target of rapamycin (mTOR) signaling and autophagy-associated genes pathway. This manuscript provides a comprehensive review of the different autophagy-mediated nanomaterials that have been developed and applied in tumor therapy. Several combination approaches for enhanced autophagy-inducing effects were also discussed.

Last but not least, the mini-review written by Linde et al. provides an overview of the recent investigations in nanoparticles strategies for improving PD-1/PD-L1 blockade-based combination therapy for triple-negative breast cancer (TNBC). In this review, the authors discussed in detail how nanotechnology can be used in combination with existing treatments. The authors concluded that nanomedicine approach represents a promising avenue for enhancing PD-1/PD-L1 blockade-based combination therapy for TNBC and encourages further development studies.

In summary, the original findings as well as critical reviews in this Research Topic have demonstrated nanomedicine as a promising strategy for developing more effective cancer combination therapies. Further extensive studies such as in-depth preclinical and clinical investigations and formulation developments, are encouraged.

## Author contributions

MP: Writing – review & editing, Writing – original draft. QM: Writing – review & editing, Writing – original draft.
